# The effect of ameloblastoma and keratocystic odontogenic tumor on the displacement pattern of inferior alveolar canal in CBCT examinations

**DOI:** 10.15171/joddd.2016.025

**Published:** 2016-08-17

**Authors:** Isa Abdi, Kourosh Taheri Talesh, Javad Yazdani, Sareh Keshavarz Meshkin Fam, Mohammad Ali Ghavimi, Seyed Ahmad Arta

**Affiliations:** ^1^Postgraduate, Department of Oral and Maxillofacial Surgery, Faculty of Dentistry, Tabriz University of Medical Sciences, Tabriz, Iran; ^2^Professor, Department of Oral and Maxillofacial Surgery, Faculty of Dentistry, Tabriz University of Medical Sciences, Tabriz, Iran; ^3^Associate Professor, Department of Oral and Maxillofacial Surgery, Faculty of Dentistry, Tabriz University of Medical Sciences, Tabriz, Iran; ^4^Assistant Professor, Department of Orthodontics, Faculty of Dentistry, Gilan University of Medical Sciences, Rasht, Iran; ^5^Assistant Professor, Department of Oral and Maxillofacial Surgery, Faculty of Dentistry, Tabriz University of Medical Sciences, Tabriz, Iran

**Keywords:** Ameloblastoma, keratocystic odontogenic tumor, mandibular canal, odontogenic tumors

## Abstract

***Background.*** The inferior alveolar canal should be examined as a significant anatomical landmark, particularly in the posterior body and ramus of the mandible, for dental and surgical procedures. In the present study, the effects of two pathological lesions, ameloblastoma and keratocystic odontogenic tumor, on canal displacement were investigated.

***Methods.*** This study had a single-blinded design. Twenty-six patients with lesions in the mandible referred to Imam Reza Hospital, Tabriz, Iran, were studied in two equal groups (13 patients with a histopathological diagnosis of ameloblastoma and 13 with a histopathological diagnosis of odontogenic keratocyst). After confirming the initial incisional biopsy and pathological report, cone beam computed tomography (CBCT) of lesions larger than 3 cm mesiodistaly and those involving the mandibular posterior body and ramus were included in the study. Two maxillofacial surgeons in association with an oral and maxillofacial radiologist examined three points on CBCT images to determine the mandibular canal position relative to the lesions from the lingual and buccal aspects.

***Results.*** The results of statistical analyses showed that in ameloblastoma, the inferior alveolar canal had been displaced more buccally in the ramus area (point A) (84.6%) but in the distal region (point C), the displacement was less buccal (41.6%). The canal was displaced buccally in 53.8% of cases at point A and in 46.2% of cases at point C in KOT lesions. Finally chi-squared test did not show any statistically significant differences between these two lesions.

***Conclusion.*** The results of this study showed no relationship between these lesions and the displacement of the mandibular canal.

## Introduction

The mandibular canal encloses the inferior alveolar nerve and vessels, which provide blood supply and innervation to the lower jaw, teeth, lips and adjacent structures.^1^Regarding sensory function and its neurovascular content, attempts to preserve the integrity of this anatomic landmark in surgical procedures can help avoid unpleasant postoperative complications and problems.^2^ The inferior alveolar canal can exhibit important anatomic variations and may be affected by inflammatory, infectious, neoplastic, iatrogenic or idiopathic lesions.^3^

Canal position assessment can be made through periapical, panoramic, CT and CBCT images. CBCT imaging technique is an important dental diagnostic imaging modality that can provide multiple projections using a cone- or pyramid-shaped beam in a single rotation.^4,5^ Images can be achieved with a lower dose due to CBCT accuracy. In addition, it has higher image quality than plain radiographs.^6^ In this study, the effects of two pathologic lesions, ameloblastoma and keratocystic odontogenic tumor, on canal displacement were reviewed.

Keratocystic odontogenic tumor may occur in any part of the jaws, with the highest prevalence in the posterior ramus and body of the mandible.^7^ Previous studies have indicated that OKC lesions can cause displacement of teeth, the inferior alveolar neurovascular bundle and other adjacent structures; they may also lead to paresthesia of the lower lip if the IAN is impinged upon. Ameloblastoma is an odontogenic tumor with a benign behavior that usually involves the posterior mandible and the third molar area.^8^ This lesion is locally destructive and may lead to IAN displacement. The inferior alveolar canal often lies adjoining to the tumor or the tumor contains the nerve.^9^

Previous studies have evaluated the mandibular canal position for placing dental implants and third molar surgeries.^10,11^ Currently only one study is available on the relationship between IAC displacement and pathologic lesions of the mandible.^12^ Therefore, the aim of this study was to evaluate the relationship between the preoperative mandibular canal position and ameloblastoma and keratocystic odontogenic tumor lesions and determine the buccolingual position associated with the lesion, making it possible for the surgeon to prepare a treatment plan.

## Methods

**Figure 1.**
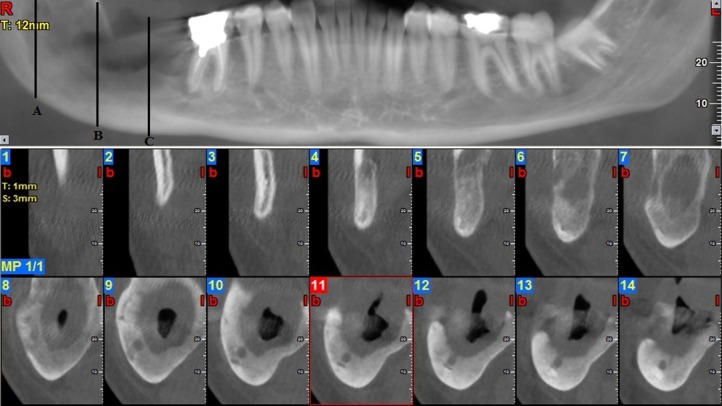
Points A, B and C with at least a 1-cm distance in a patient with ameloblastoma with buccal displacement.

Ethical approval was obtained from the Ethics Committee of Tabriz University of Medical Sciences (Ref. No. 5/4/3816). The present investigation included the patients who had referred to Imam Reza Hospital, an educational/treatment center, with lower jaw lesions, from 2014 to 2016. The patients were included after obtaining informed consent. The patients with a diagnosis of ameloblastoma and keratocystic odontogenic tumor lesions, affecting the posterior body and mandibular ramus, in the initial differential diagnosis were selected. The patients enrolled in the study had lesions histopathologically diagnosed as ameloblastoma and KOT. At first, an incisional biopsy of the lesion was carried out under local anesthesia. The biopsy samples were taken from different areas in cases where the lesion was large, so that the minimum mesiodistal diameter of each lesion was at least 3 cm. Patients with a history of previous trauma at the site of the lesion, congenital deformity and lesion recurrence, were excluded. After histopathological confirmation of each lesion, the CBCT evaluation of these patients was provided. All the CBCT scans were performed with NEWTOM VGI machine. In the CBCT scans, three points from the ramus to the body of the mandible were designated as points A, B and C. Point A was examined first and included the mandibular canal in relation to the lesion in the ramus where the mandibular canal and the lesion in the ramus were distinguishable. Point B was considered a more distal than point A with a 1-cm distance in the body and point C was more distal than point B at a distance of 1 cm (Figure 1).

Two oral and maxillofacial surgeons along with a maxillofacial radiologist separately assessed the mandibular canal position to determine lingual and buccal displacements (Figure 1 The investigators were blinded to the results of the histopathological examination and the other investigators’ opinions. Cases with undistinguishable mandibular canal borders were excluded. First, two practitioners independently determined the relationship of the canal with the lesion at all three points. Displacement was recorded as buccal, lingual and no displacement (Table 1). The final position in borderline cases was determined by a third examiner after assessments made by two former examiners; after the position was confirmed by two examiners, it was recorded as a final result.

**Table 1:** Inferior alveolar canal displacement in ameloblastoma and keratocystic odontogenic tumor (KOT) in relation to lesion

**Pt. No**	**Lesion type**	**IAN displacement on review of CBCT**		
		**Point A**	**Point B**	**Point C**
**1**	Ameloblastoma	Buccal	buccal	No displacement
**2**	Ameloblastoma	Buccal	buccal	buccal
**3**	Ameloblastoma	Buccal	buccal	Buccal
**4**	Ameloblastoma	Lingual	lingual	Lingual
**5**	Ameloblastoma	Lingual	lingual	Lingual
**6**	Ameloblastoma	Buccal	buccal	Buccal
**7**	Ameloblastoma	Buccal	buccal	Buccal
**8**	Ameloblastoma	Buccal	buccal	Buccal
**9**	Ameloblastoma	Buccal	No displacement	Lingual
**10**	Ameloblastoma	Buccal	buccal	Buccal
**11**	Ameloblastoma	Buccal	buccal	No displacement
**12**	Ameloblastoma	Buccal	buccal	Lingual
**13**	Ameloblastoma	Buccal	No displacement	No displacement
**14**	KOT	Lingual	lingual	Lingual
**15**	KOT	Lingual	lingual	Lingual
**16**	KOT	Buccal	buccal	Buccal
**17**	KOT	Lingual	lingual	Lingual
**18**	KOT	Lingual	lingual	Lingual
**19**	KOT	Lingual	lingual	Lingual
**20**	KOT	Buccal	buccal	Buccal
**21**	KOT	Buccal	buccal	Buccal
**22**	KOT	Buccal	buccal	Lingual
**23**	KOT	Lingual	lingual	No displacement
**24**	KOT	Buccal	buccal	No displacement
**25**	KOT	Buccal	buccal	Buccal
**26**	KOT	Buccal	buccal	Buccal

Finally, 26 patients referred to the hospital, of which 13 had keratocystic odontogenic tumor and 13 had ameloblastoma, were examined in the study.

## Results

Of 13 patients with histological diagnosis of ameloblastoma, 6 patients were female and 7 were male, with an age range of 16‒63 years and of the 13 patients with histological diagnosis of keratocystic odontogenic tumor (KOT), 5 were female and 8 were male, with an age range of 16‒53 years.

At point A (the most proximal point), the results of statistical analysis showed that 84.6% of ameloblastoma lesions led to buccal and 15.4% led to lingual displacement of the inferior alveolar canal. Of all KOT lesions, 53.8% led to buccal displacement and 46.2% led to lingual displacement of the IAC. However, chi-squared test showed no statistically significant differences (P = 0.08; Table 2).

**Table 2:** Displacement at point A in ameloblastoma and keratotic odontogenic tumor (KOT)

**Lesion type**	**IAC displacement type**		
	**Buccal**	**No Displacement**	**Lingual**
**Ameloblastoma**	11	0	2
	84.6%	0%	15.4%
**KOT**	7	0	6
	53.8%	0%	46.2%

At point B, the results showed that 69.2% of ameloblastoma lesions resulted in buccal displacement, 15.4% exhibited no displacement and 15.4% resulted in lingual displacement of the IAC. In addition, 53.8% of KOT lesions led to buccal displacement and 46.2% led to IAC lingual displacement as well. However, chi-squared test did not show any statistically significant differences (P = 0.11; Table 3).

**Table 3:** Displacement at point B in ameloblastoma and keratotic odontogenic tumor (KOT)

**Lesion type**	**IAC displacement type**		
	**Buccal**	**No Displacement**	**Lingual**
**Ameloblastoma**	9	2	2
	69.2%	15.4%	15.4%
**KOT**	7	0	6
	53.8%	0%	46.2%

**Figure 2.**
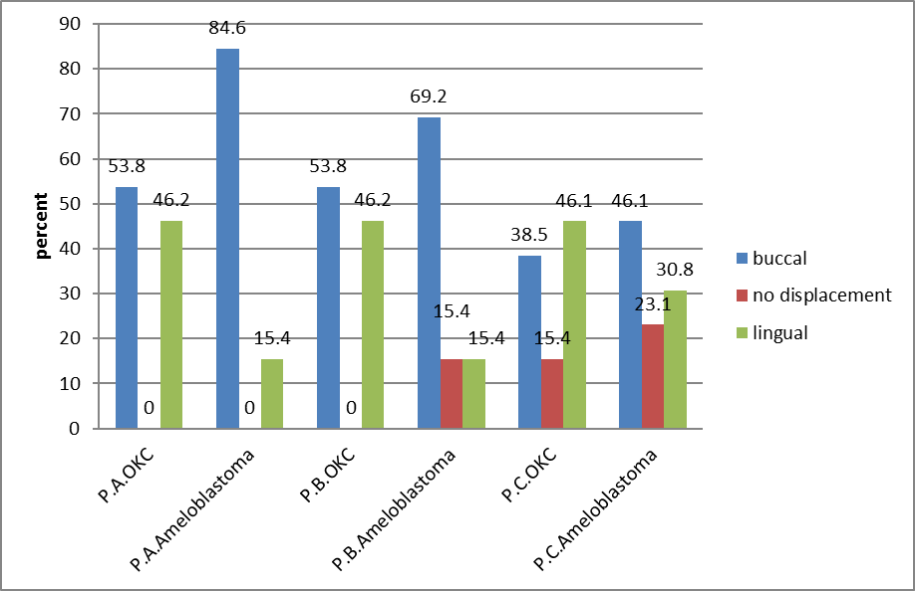
Comparison of displacement of points A, B and C in ameloblastoma and keratotic odontogenic tumor. P.A.OKC: point A in keratocyst odontogenic tumor; P.B.OKC: point B in keratocyst odontogenic tumor; P.C.OKC: point C in keratocyst odontogenic tumor; P.A.Ameloblastoma: point A in ameloblastoma; P.B.Ameloblastoma: point B in ameloblastoma; P.C.Ameloblastoma: point C in ameloblastoma; Point A: The firs point that was evaluated in CBCT; Point B: The second point that was evaluated in CBCT; Point C: The third point that was evaluated in CBCT.

At point C (the most distal IAC assessment point located in the mandibular body ), the results showed that 41.1% of ameloblastoma lesions resulted in buccal displacement, 23.1% resulted in no displacement and 30.8% resulted in lingual displacement of the IAC. Also, 38.5% of KOT lesions led to buccal displacement, 15.4% resulted in no displacement and 46.1% led to lingual displacement of the IAC. Chi-squared test did not show any statistically significant differences (P=0.70) (Table 4). Therefore, the statistical analyses showed that no relationship between these lesions and the inferior alveolar canal displacement (Figure 2).

**Table 4:** Displacement at point C in ameloblastoma and keratotic odontogenic tumor (KOT)

**Lesion type**	**IAC displacement type**		
	**Buccal**	**No Displacement**	**Lingual**
**Ameloblastoma**	6	3	4
	46.1%	23.1%	30.8%
**KOT**	5	2	6
	38.5%	15.4%	46.1%

## Discussion

The course of the inferior alveolar canal is of utmost significance in the dental practice and surgical procedures because this anatomical landmark influences the inferior alveolar nerve anesthetic block, tooth extractions, the planning of surgeries for pathologic lesions and for orthognathic purposes, for placing and determining dental implant sizes and dimensions of the grafts removed from the ramus and donor sites.^13-15^

The inferior alveolar canal is a route starting from the medial ramus to the mental foramen in the mandibular body.^6^ The route of the canal within bone creates an S-shaped pattern; this is where the canal is most lateral in the third molar area and closest to the buccal cortex. It then approaches the lingual plate in the first molar area. Anteriorly, between the premolars, the IAC returns toward the buccal cortex prior to exiting the mandible through the mental foramen. In the sagittal plane, the course of the inferior alveolar nerve in the mandible begins superiorly and lingually at the lingula, approximately 10 mm from the sigmoid notch and reaches its lowest point at the region of first molar to second premolar, with a distance of 7.5±1 mm from the mandibular inferior border, where it splits into the incisive and mental nerve branches.^16^

It has been demonstrated that the distance between the mandibular canal and the buccal cortex is greater in the molar region than in the ramus region.^17^ This distance exhibits high variations in measurements from 0.57 to 5.8 mm in the ramus region and from 0.40 to 7.0 mm in the molar region.^18^ This great diversity is due to various studies on different races, age groups and different anatomical variations, which also holds true for other anatomic structures. The posterior mandible and ramus are among the areas affected by pathologic lesions that have been considered in this study. In our study, two pathologic lesions, ameloblastoma and keratocystic odontogenic tumor, which occur most commonly in the posterior mandible tooth-bearing area, were studied to determine the mandibular canal position in different preoperative assessments.

Ameloblastoma is the most common benign odontogenic tumor with an origin of epithelium to affect the mandible,^19^ accounting for about 1% of all tumors and cysts involving the jaws. Approximately 80% of ameloblastomas occur in the lower jaw, mainly in the third molar region.^20^

Odontogenic keratocysts have recently been introduced as keratocystic odontogenic tumors characterized by stratified squamous epithelium.^7^ They are one of the most aggressive odontogenic lesions, and tend to invade adjacent tissues with a high recurrence rate. The posterior mandibular and third molar areas are the most common sites for these cysts.

Given the prevalence of site and the growing nature of these lesions, assessing the relevance and potential impact of these two lesions on the IAC, which is an important anatomical structure, seems logical.

Several studies have examined the anatomy of the IAC using different radiographic techniques, CT scans and other methods.^2,8,21,22^ Since CBCT imaging is capable of measuring and determining exact details better than plain radiography and standard CT, it was used in this study to evaluate canal displacement. It is possible to separate the mandibular canal anatomy from lesions by CBCT with greater certainty due to multiple projections with a single rotation^1,23^ in comparison with panoramic, periapical and CT scans. The standard CT protocol was retrospectively studied to review the effect of odontogenic tumors and vascular lesions on canal displacement in a study.^24^ A CBCT scan can show minimal metal artifacts and can be handled easily with lower costs^17^ and its use imposes a lower radiation dose on the patient than conventional CT scans. The evaluation system makes the three-dimensional evaluation of bony structures possible, with greater accuracy, compared with plain radiographs.^23,25^

In reviewing the results, buccal canal displacement in point A (in the ramus region) was more common (84.61%) compared to points B and C in patients with mandibular lesions of ameloblastoma. This may indicate that the initial route of the canal was influenced during the growth of the lesion. Based on what was mentioned above, the mandibular canal is more buccal in the ramus and posterior third molar area than in anterior regions. This is due to the origin of ameloblastoma which is an odontogenic lesion and is more likely to push the canal to the buccal aspect of its origin (dental follicle epithelium which is generally more lingual than the canal of mandible). But in points B and C of the mandibular body, the canal assumes a more lingual direction and the possibility of buccal displacement decreases. Investigation of keratocystic odontogenic tumor showed no significant difference in lingual or buccal displacement of the IAC. This can be explained by the invasive nature of this lesion, which affects adjacent bony structures with minimal anatomical structure displacement, such as teeth, IAC and cysts around the canal. Thus the canal can accidentally be displaced buccally or lingually.

Although some studies have studied the growth characteristics of mandibular pathologic lesions like ameloblastoma and odontogenic tumours,^9,26^ these studies have not described the relationship between these pathologic lesions and the position of the canal. Kolokythas et al^12^ examined the potential difference in the displacement of inferior alveolar neurovascular bundle caused by odontogenic tumors and vascular anomalies. Their study consisted of 13 ameloblastomas, 2 odontogenic myxomas and one ameloblastic fibroma and 8 lesions with vascular origin. The study showed significant differences in IAC displacement between odontogenic tumors and vascular anomalies. They concluded that neoplasms of odontogenic origin tend to displace the IAC buccally at points A, B and C, without any lingual displacement, and vascular anomalies tend to displace the IAC toward the lingual cortex in all of the lesions. Kolokythas study was a retrospective review of CT images. The inferior alveolar canal was traced from the lingula to the mental foramen and they recorded displacement of IAC at three points. In contrast to Kolokythas study, in our study, lingual displacement was recorded specially at points B and C, which is on the course of IAN, as previously described. Also in the present study, CBCT was used instead of CT as a more precise modality with lower radiation dose.

Due to the sample size of the study, studies with larger sample sizes are recommended. In addition, it is suggested that the effects of other common cysts and tumors on adjacent anatomical landmarks be investigated. Since previous studies have shown variations in the inferior alveolar canal position with age and gender, it seems logical that these criteria should be considered.^17-21^

## Acknowledgments

The financial support of Research Deputy of Tabriz University of Medical Sciences is gratefully acknowledged.

## Authors’ contribution

IA, KTT, MAG, SKMF, JY, and SAA contributed to the design of the study and definition of the intellectual content. IA performed the literature search and review and the experimental studies, and drafted the manuscript. IA, KTT, and MAG were responsible for all the aspects of the work in ensuring that all parts of the work were accurately and appropriately investigated. All the authors contributed to critical revision of the manuscript, and have read and approved the final manuscript.

## Funding

This research was financed by Vice Chancellor for Research of Tabriz University of Medical Sciences.

## Competing interests

The authors declare no competing interests with regards to the authorship and/or publication of this article.

## Ethics approval

The study was approved by the Ethics Committee of Tabriz University of Medical Sciences (Ref. No. 5/4/3816).
